# Aerobic Exercise Program Management Enhances Mental and Physical Health in Overweight/Obese Adolescents With Depression: Insights From a Retrospective Study

**DOI:** 10.62641/aep.v53i3.1995

**Published:** 2025-05-05

**Authors:** Jian Wang, Hong Zhang

**Affiliations:** ^1^Psychological Trauma Ward, Wuhan Mental Health Center Affiliated to Tongji Medical College of Huazhong University of Science and Technology, 430030 Wuhan, Hubei, China

**Keywords:** overweight/obese adolescents, depression, aerobic exercise, quality of life

## Abstract

**Background:**

Overweight/obese adolescents show higher rates of depression. This study aims to explore the effect of aerobic exercise program management on overweight/obese adolescents with depression.

**Methods:**

This retrospective study analyzed clinical data of overweight/obese adolescents with depression at Wuhan Mental Health Center, from June 2019 to June 2022. Propensity score matching (PSM), *t*-tests, and chi-square tests were applied. Observation indexes including Hamilton Anxiety Scale (HAMA), Hamilton Depression Rating Scale 17 (HAMD-17), Simplified Coping Style Questionnaire (SCSQ), body mass index (BMI), waist–hip ratio (WHR), nursing effectiveness, and Generic Quality of Life Inventory-74.

**Results:**

A total of 229 depressed overweight/obese adolescents were divided into two groups: the control group (n = 108) receiving routine care only, and the observation group (n = 121) receiving routine care along with aerobic exercise. After 1:1 PSM (caliper 0.02), groups (each group comprised 104 patients) showed no baseline differences. No significant differences were found in HAMA, HAMD-17, SCSQ, quality of life scores, BMI, and WHR pre-exercise (*p* > 0.05). Post-nursing care, the observation group exhibited significantly lower HAMA (*t* = 3.589, *p* = 0.001), HAMD-17 (*t* = 3.554, *p* = 0.001), SCSQ negative scores (*t* = 3.584, *p* = 0.001), BMI (*t* = 3.051, *p* = 0.003), and waist-to-hip ratio (*t* = 3.379, *p* = 0.001), with higher SCSQ positive (*t* = 3.443, *p* = 0.001) and quality of life in material life (*t* = 3.385, *p* = 0.001), physical function (*t* = 3.834, *p* < 0.001), social function (*t* = 3.485, *p* = 0.001), psychological function (*t* = 3.178, *p* = 0.002) compared to the control group.

**Conclusion:**

The aerobic exercise program care for overweight/obese adolescents with depression, which is advocated and has a high nursing effect, can effectively improve the anxiety and depression of patients.

## Introduction

Long-term excess energy intake and consumption will lead to excessive fat accumulation in the body, which eventually manifests as overweightness/obesity [1]. Given unreasonable diet, excessive dietary structure, reduced exercise, and genetic and mental factors [2, 3], certain teenagers are likely to be overweight/obese. With the improvement of physical health, stature, and other aspects, adolescents are increasingly becoming concerned about overweightness/obesity, which may gradually translate into psychological problems, which manifests itself in significant and prolonged cases of depression such as irritability, low mood and emotional instability [4]. Compared with their normal-weight peers, overweight/obese adolescents are more likely to have psychological behavior problems and show a higher incidence of depression [5, 6]; such condition affects adolescents’ health and psychological well-being and their way of doing things, which are very unfavorable to their growth [7]. Consequently, scientific and effective conditioning nursing care is needed for such patients. Referring to previous research findings, it is recognized that physical exercise is an essential part of the prevention and treatment of depression, as well as proven to be able to reduce depressive symptoms and schizophrenia [8, 9]. Moreover, Liu X and other scholars [10] have demonstrated through further research that aerobic exercise in physical exercise can significantly improve the mood of patients with major depression in a short period of time, especially effective for mild-to-moderate depression. Aerobic exercise program nursing is through the combination of the patient’s actual physical condition and psychological problems, to develop a corresponding exercise plan for the patient. Thus, through the way of aerobic exercise to improve the body index and regulate the psychological condition, it has been commonly used in the clinic and achieved better results [11, 12, 13, 14].

Although previous studies [15, 16, 17, 18] have shown that aerobic exercise can improve depression, research on the management of depression in overweight/obese adolescents through aerobic exercise is limited. Additionally, understanding of specific management strategies and their actual effects on patients remains limited. This retrospective analysis of overweight/obese adolescent depression patients explores the correlation between aerobic exercise program management and patients’ emotions, quality of life, and coping mechanisms, thus gaining deeper insights into the potential physiological and psychological effects of aerobic exercise on patients. Unlike previous studies, this research applies aerobic exercise program management to the specific population of overweight/obese adolescents, representing an innovative endeavor. In comparison to traditional treatment methods such as medication or generalized psychological interventions, this study proposes a more targeted treatment approach, offering new perspectives and methods for managing depression in this specific population. Aerobic exercise program management, as a comprehensive treatment strategy, combines the features of exercise and mental health, offering unique advantages. Compared to singular treatment modalities, the treatment approach in this study is more holistic, capable of simultaneously improving patients’ physical and mental health statuses, thus providing a novel approach and avenue for the treatment of depression in overweight/obese adolescents.

## Materials and Methods

### Research Object

A total of 229 overweight/obese adolescents with depression and admitted to the department of psychiatry and psychology of Wuhan Mental Health Center from June 2019 to June 2022 were included in this study. Depending on the different types of nursing care recorded in the medical record system, it was divided into a control group (n = 108, routine nursing care), and an observation group (n = 121, routine nursing care + aerobic exercise program). The inclusion criteria were as follows: (1) body mass index (BMI) of 24 kg/m^2^ or higher; (2) age 13–19 years old; (3) confirmed depression in accordance with the diagnostic and statistical manual of mental disorders in diagnostic and statistical manual of mental disorders, 5th edition [19]; (4) complete clinical data; (5) course of the disease lasting more than 1 month and showing a stable condition; (6) normal cardiovascular function and ability to withstand load movement. The exclusion criteria included the following: (1) presence of severe organic brain lesions; (2) serious physical diseases; (3) conditions merging with other serious mental illness; (4) poor communication skills and inability to complete psychological assessment; (5) electroconvulsive therapy within the past 1 month; (6) incomplete involvement. The study was approved by the ethics committee of Wuhan Mental Health Center (No: KY2019.0522.01). Informed consent was required and obtained in this work. This study was performed in accordance with the principles of the Declaration of Helsinki.

### Methods

#### Control Group

Patients in the control group received routine nursing care for 2 months: (1) The nursing staff explained and publicized routine health contents, such as disease knowledge, safety education, psychological care, and drug guidance; (2) nurses answered patients’ queries in a timely manner, guided patients in conversations, understood and mastered patients’ psychological needs, and encouraged patients when they performed well; (3) nurses explained the importance of family accompany and support, guided family members to analyze the problems of patients with their family, and put forward their own opinions depending on the problem; (4) administration of escitalopram antidepressant and citalopram tablets (Jilin Province West Pharmaceutical Technology Development Co., Ltd., Changchun, Jilin, China; 5 mg, batch number: H20140108) approved by the state and sertraline hydrochloride (Beijing New Pharmaceutical Co., Ltd., Hangzhou, Zhejiang, China; 50 mg, batch number: H20051076). 5 mg/day and a maximum dose of 20 mg/day for the initial dose of escitalopram tablets; 50 mg/day and a maximum dose of 200 mg/day for sertraline hydrochloride tablets; (5) a WeChat group was established with the patient’s family members, in which the patient and his/her family members are included. Every Wednesday and Friday, the group regularly pushes disease-related treatment content and precautions, as well as urging the patient’s family members to provide feedback on the patient’s situation every Sunday. In case of abnormalities, the patient was promptly admitted to the hospital for diagnosis and treatment, meanwhile, the patient’s family was reminded to bring the patient to the hospital for review every 2 weeks.

#### Observation Group

Patients in the observation group were cared for with a 2-month aerobic exercise program based on usual nursing care, as shown in Table 1.

**Table 1.** **Aerobic exercise care breakdown**.

Step	Specific nursing care processes
(1) Programming	To recognize the patient’s daily interests, a variety of aerobic exercise modalities such as fitness running, rope skipping, badminton, table tennis, basketball, etc., were mainly chosen. The fitness program is developed according to each patient’s different body mass index and exercise tolerance.
(2) Selection of aerobic exercise types and heart rate control	① Fitness running was performed at 5–6 pm every day, with the running speed controlled at 120 steps/min, and the time for each session was 40–50 min; ② The patients wore a heart rate monitor when exercising, with the heart rate controlled at 70%–80% of the maximal heart rate (maximal heart rate = 220 – age), and the exercise was performed 4–5 times per week.
(3) Exercise guidance	To explain the effects of aerobic exercise to patients and their families. It is emphasized that aerobic exercise is valuable for patients in terms of psychological adjustment, improvement of depression, and change in body mass, with examples of successful cases to support this. As a result, it contributes to increasing the trust and cooperation of patients and their families, which in turn enhances the patients’ motivation to engage in aerobic exercise.
(4) Conducting fun games	Each exercise program is completed by 4∼6 patients collaboratively. Patients voluntarily team up with each other to participate, and patients are encouraged to share their feelings after the end of the program.
(5) Relaxation training	After the patient rested until calm, soothing music was played and the patient was instructed to perform 1 min of breathing exercises to maintain a relaxed state. Patients were instructed to perform breathing exercises and meditation for relaxation after each exercise.

### Observation Indicators

The scores on Hamilton Anxiety Scale (HAMA), Hamilton Depression Rating Scale 17 (HAMD-17), Simplified Coping Style Questionnaire (SCSQ), BMI, waist–hip ratio (WHR), nursing effect, and General Quality of Life Inventory 74 (GQOLI-74) were compared between the two groups after matching.

(1) The patients were evaluated using their scores on HAMA, which consists of 14 items, with each item scored 1–4 points, and the total score is 56. The more severe the anxiety, the higher the score, with a Cronbach’s alpha coefficient of 0.860. The scores on HAMD, which contains 17 items, were obtained to evaluate the patients; score <7 was considered normal; 7–17, mild depression; 18–24, medium depression; >24, major depressive disorder; the higher the score, the more depressing the mood; Cronbach’s alpha coefficient was 0.915 [20, 21].

(2) The SCSQ score includes two dimensions: positive and negative coping, each of which had a score of 0–30 and a total score of 60. The higher the score in the coping style, the more inclined the patients to adopt the coping style. The Cronbach’s α coefficient was 0.841 [22].

(3) The BMI and WHR of patients before and after nursing care were detected and compared.

(4) The GQOLI-74 scale includes four dimensions: material life, physical function, social function, and psychological function; the total score of each dimension was 100, and the higher the score, the better the quality of life of patients; Cronbach’s α coefficient was 0.881 [23, 24].

### Statistical Methods

All statistical analyses in this study were statistically presented using IBM SPSS Statistics for Windows version 27.0 (IBM Corp., Armonk, NY, USA). Measurements that conformed to normal distribution were expressed as ($\bar{x}$ ± s), while those that did not conform to normal distribution were statistically analyzed after the variables were transformed to normal distribution. The *t*-test, comparison of continuous variables between two groups, count data, expressed as [n (%)], were compared using the χ^2^ test, with a two-tailed *p*-value of <0.05 as the threshold of statistical significance. Selecting age, gender, grade, height, family economic status, duration of illness, residence, being an only child, family history of mental illness, and level of depression as matching variables, the values of propensity score were calculated by logistic regression analysis. The control group and the observation group were unfolded to be matched according to 1:1 ratio nearest neighbor matching method, with caliper value taken as 0.02 [25]. Effect size was represented using Cohen’s d. It is calculated by dividing the difference between the means of two groups by the pooled standard deviation of the two groups [26, 27]. A Cohen’s d value of 0.2 was considered a small effect; 0.5 was considered a medium effect; while 0.8 or higher was considered a large effect [28].

## Results

### Comparison of General Data Between the Two Groups

A total of 229 patients with depression in overweight/obese adolescents were included in this study. Based on different care modalities, it was divided into a control group (n = 108) and an observation group (n = 121). There was no significant difference in general information such as age, gender, grade, height, family economic status, duration of illness, residence, being an only child, family history of mental illness, degree of depression and medication use between the two groups after matching (*p* > 0.05), so they are comparable, see Table 2.

**Table 2.** **Comparison of general data**.

Indicators		Before matching				After matching			
		Control group (n = 108)	Observation group (n = 121)	χ^2^/*t*	*p*	Control group (n = 52)	Observation group (n = 52)	χ^2^/*t*	*p*
Age (years, $\bar{x}$ ± s)		16.75 ± 1.68	15.22 ± 1.53	7.213	<0.001	15.56 ± 1.57	15.60 ± 1.58	0.130	0.897
Gender (n, %)	Male	60 (55.56)	61 (50.41)	0.606	0.436	28 (53.85)	30 (57.69)	0.156	0.693
	Female	48 (44.44)	60 (49.59)			24 (46.15)	22 (42.31)		
Grade (n, %)	Junior high school	59 (54.63)	65 (53.72)	0.019	0.890	27 (51.92)	29 (55.77)	0.155	0.694
	High school	49 (45.37)	56 (46.28)			25 (48.08)	23 (44.23)		
Height (cm)		158.75 ± 15.87	161.2 ± 16.21	1.153	0.250	156.33 ± 16.11	156.15 ± 16.15	0.057	0.955
Family economic status (n %)	Preferably	32 (29.63)	39 (32.23)	0.186	0.911	14 (26.92)	15 (28.85)	0.301	0.860
	Normal	56 (51.85)	60 (49.59)			29 (55.77)	30 (57.69)		
	Poor	20 (18.52)	22 (18.18)			9 (17.31)	7 (13.46)		
Duration of disease (years)		1.03 ± 0.50	1.18 ± 0.51	2.242	0.026	1.08 ± 0.31	1.10 ± 0.30	0.334	0.739
Residence (n, %)	Rural areas	35 (32.41)	40 (33.06)	0.011	0.917	16 (30.77)	18 (34.62)	2.927	0.087
	City	73 (67.59)	81 (66.94)			36 (69.23)	34 (65.38)		
Only child or not (n, %)	Yes	67 (62.04)	77 (63.64)	0.063	0.803	31 (59.62)	33 (63.46)	0.163	0.687
	No	41 (37.96)	44 (36.36)			21 (40.38)	19 (36.54)		
Family history of mental illness (n, %)	Yes	18 (16.67)	22 (18.18)	0.091	0.763	4 (7.69)	6 (11.54)	0.443	0.506
	None	90 (83.33)	99 (81.82)			48 (92.31)	46 (88.46)		
Degree of depression (n,)	Mild	45 (41.67)	48 (39.67)	0.097	0.953	23 (44.23)	25 (48.08)	0.163	0.922
	Medium	36 (33.33)	42 (34.71)			19 (36.54)	18 (34.62)		
	Severe	27 (25.00)	31 (25.62)			10 (19.23)	9 (17.31)		
Escitalopram tablets	Initial average dose (mg/d)	5.65 ± 0.57	5.70 ± 0.58	0.657	0.512	5.67 ± 0.57	5.68 ± 0.58	0.089	0.930
	Average maximum dose (mg/d)	17.65 ± 1.88	17.58 ± 1.76	0.291	0.771	17.60 ± 1.76	17.62 ± 1.77	0.058	0.954
Sertraline hydrochloride tablets	Initial average dose (mg/d)	56.75 ± 5.69	56.85 ± 5.70	0.133	0.895	56.78 ± 5.68	56.82 ± 5.69	0.036	0.972
	Average maximum dose (mg/d)	158.75 ± 16.88	159.10 ± 16.90	0.157	0.876	158.90 ± 16.01	158.95 ± 16.12	0.016	0.987

### Comparison of Psychological Status Scores

There was no significant difference in HAMA and HAMD-17 scores between the two groups before nursing care (*p* > 0.05, Table 3), and the effect size were 0.024 and 0.016, which was a small effect. While each score was lower in the observation group compared to the control group after nursing care (*t* = 3.589, *p* = 0.001, Table 3), (*t* = 3.554, *p* = 0.001, Table 3), and the effect size were 0.704 and 0.694, which was a medium effect.

**Table 3.** **Comparison of HAMA and HAMD-17 scores (scores, $\bar{x}$ ± s)**.

Group	n	HAMA		HAMD-17	
		Before nursing care	After nursing care	Before nursing care	After nursing care
Observation group	52	35.37 ± 4.58	16.73 ± 3.22	24.65 ± 2.53	15.55 ± 2.56
Control group	52	35.48 ± 4.67	19.19 ± 3.75	24.61 ± 2.51	17.43 ± 2.85
*t*	-	0.122	3.589	0.081	3.554
*p*	-	0.903	0.001	0.935	0.001

HAMA, Hamilton Anxiety Scale; HAMD-17, Hamilton Depression Rating Scale 17.

### Comparison of SCSQ Scores

There was no significant difference in SCSQ scores between the two groups before nursing care (*p* > 0.05, Table 4), and the effect size were 0.071 and 0.010, which was a small effect. The positive coping dimension scores were higher in the observation group than in the control group after nursing care (*t* = 3.443, *p* = 0.001, Table 4) while the negative coping dimension scores were lower in the observation group than in the control group (*t* = 3.584, *p* = 0.001, Table 4), and the effect size were 0.672 and 0.700, which was a medium effect.

**Table 4.** **Comparison of SCSQ scores (scores, $\bar{x}$ ± s)**.

Group	n	Respond actively		Negative coping	
		Before nursing care	After nursing care	Before nursing care	After nursing caret
Observation group	52	8.27 ± 0.85	16.79 ± 3.12	19.60 ± 2.01	6.75 ± 1.78
Control group	52	8.21 ± 0.84	14.73 ± 3.01	19.58 ± 2.05	8.02 ± 1.85
*t*	-	0.363	3.443	0.051	3.584
*p*	-	0.716	0.001	0.960	0.001

SCSQ, Simplified Coping Style Questionnaire.

### Comparison of BMI and WHR

Before nursing care, the BMI and WHR ratio of the two groups were the same (*p* > 0.05, Table 5), and the effect size were 0.035 and 0.074, which was a small effect. After nursing care, the observation group showed lower BMI (*t* = 3.051, *p* = 0.003, Table 5) and WHR (*t* = 3.379, *p* = 0.001, Table 5) than the control group, and the effect size were 0.600 and 0.661, which was a medium effect.

**Table 5.** **Comparison of BMI and WHR ($\bar{x}$ ± s)**.

Group	n	BMI (kg/m^2^)		WHR (cm)	
		Before nursing care	After nursing care	Before nursing care	After nursing care
Observation group	52	28.21 ± 2.83	24.85 ± 2.46	1.35 ± 0.14	0.72 ± 0.22
Control group	52	28.11 ± 2.82	26.37 ± 2.64	1.34 ± 0.13	0.89 ± 0.29
*t*	-	0.181	3.051	0.379	3.379
*p*	-	0.856	0.003	0.705	0.001

BMI, body mass index; WHR, waist–hip ratio.

### Comparison of Quality of Life Scores

Before nursing care, the quality of life scores of the two groups were the same (*p* > 0.05, Table 6), and the effect size were 0.006, 0.060, 0.070 and 0.006, which was a small effect. After nursing care, the observation group exhibited higher scores than the control group in material life (*t* = 3.385, *p* = 0.001, Table 6), physical function (*t* = 3.834, *p* < 0.001, Table 6), social function (*t* = 3.485, *p* = 0.001, Table 6), psychological function (*t* = 3.178, *p* = 0.002, Table 6), and the effect size were 0.660, 0.748, –0.680 and –0.620, which was a medium effect.

**Table 6.** **Comparison of quality of life scores (scores, $\bar{x}$ ± s)**.

Group	n	Material life		Physical function		Social function		Psychological function	
		Before nursing care	After nursing care	Before nursing care	After nursing care	Before nursing care	After nursing care	Before nursing care	After nursing care
Observation group	52	65.23 ± 6.61	81.42 ± 8.12	64.21 ± 6.48	78.25 ± 8.02	61.37 ± 6.21	84.10 ± 8.51	64.31 ± 6.48	83.25 ± 8.35
Control group	52	65.27 ± 6.58	76.21 ± 7.65	64.58 ± 6.52	72.52 ± 7.28	61.79 ± 6.20	78.52 ± 7.89	64.35 ± 6.51	78.21 ± 7.91
*t*	-	0.031	3.385	0.291	3.834	0.347	3.485	0.032	3.178
*p*	-	0.975	0.001	0.771	<0.001	0.729	0.001	0.975	0.002

## Discussion

This study aimed to investigate the effects of aerobic exercise program management on depression in overweight/obese adolescents and drew a series of significant conclusions through retrospective analysis. The results revealed a notable improvement in depression symptoms among this specific population with the implementation of aerobic exercise program management. Specifically, it was found that augmenting conventional care with aerobic exercise program management effectively alleviated anxiety and depressive moods in the patients. Furthermore, this management approach led to significant reductions in the patients’ BMI and waist-to-hip ratio, improved their social functioning, and enhanced their quality of life. Importantly, this management modality also facilitated changes in how patients dealt with problems. These findings underscore the potential value of aerobic exercise as a non-pharmacological treatment method for managing depression in overweight/obese adolescents and provide crucial theoretical and practical guidance for clinical practice.

In this study, the SCSQ scores of the two groups were the same before nursing care. After nursing care the observation group exhibited higher and lower positive and negative coping dimension scores, respectively, than the control group. These findings indicate a possible correlation between patient matter coping styles and aerobic exercise. Gilbertson NM *et al*. [29] reported that aerobic exercise can increase a patient’s brain blood circulation, release of endorphins and monoamine, and activation of greater hypothalamic–pituitary–adrenal axis and improve one’s manner of handling of things. The above findings are consistent with those in the present study. Van de Pas KGH *et al*. [30] showed that overweight or obese adolescents with depression were discriminated against and bullied due to their weight, which led to their negative perception of self-image and dissatisfaction with their body; this condition resulted in emotional instability, anxiety, and negative coping styles. In this study, the daily interests of patients in the observation and control groups learned were determined. A variety of aerobic exercise methods, such as fitness running, rope skipping, badminton, table tennis, basketball, and so on, were selected, and plans were made based on the actual physical quality and disease needs of the patients. Thus, the patients can effectively express and stabilize their negative emotions in the form of aerobic exercise. Through engagement in interesting sports, strengthening patient group cooperation, and improving patients’ social function, the value of aerobic exercise for patients was emphasized, and successful cases were given as evidence to improve patients’ enthusiasm for aerobic exercise, change their attitude toward the disease, motivate them to face depression in a positive manner, and improve their willpower, collective concept, competition consciousness, and interpersonal communication through scientific and effective exercise methods to give full play to the patients’ subjective initiative.

The data obtained in this study show that the HAMA and HAMD scores of the two groups were the same before nursing care, and the scores of the observation group became lower than those of the control group afterward. Before nursing care, the quality of life scores of the two groups were the same. After nursing care, the observation group showed higher scores than the control group, which indicates that aerobic exercise nursing care can effectively reduce anxiety and depression and improve the quality of life. Zaragas H *et al*. [31] were consistent with those of this study. Aerobic exercise, in terms of the patients’ attention, self-control, planning, decision making, execution, and other aspects, effectively reduced the level of anxiety and depression and improve their quality of life. The quality of life indicates that the patient’s subjective feelings regarding all aspects of life, including material life, physical function, and other aspects, can effectively reflect their physiological, psychological, and social adaptation state; a patient’s psychological state decreases with the decrease in the quality of life. Chalise A *et al*. [32] believed that patients with depression will lose enthusiasm and fun in life, usually feel depressed and lonely, and have low energy, which result in anxiety. In addition, such conditions can cause difficulty in focusing attention, memorization, and thinking, which cause patients to inhibit from communicating with their friends and family and refuse to participate in social activities, which affect their quality of life. In this study, based on the actual situation of patients in the observation group, the aerobic exercise program was formulated, an interesting sports meeting was held, and the patients were guided to carry out breathing training, meditation, and relaxation after exercise nursing care, which effectively relieved their emotions, changed their brain chemical composition, improved their memory and attention, and effectively increased their quality of life.

The data obtained this study show that the BMI and WHR of the two groups were the same before nursing care, and the values for the observation group were lower than those for the control group afterward, which indicate that aerobic exercise can substantially improve the BMI and WHR of patients. Lidaka L *et al*. [33] also observed that appropriate exercise nursing care for overweight or obese adolescents can effectively improve their BMI. Aerobic exercise can change the activities of fat metabolism enzymes and promote fat metabolism and utilization.

This study has certain clinical significance. Through clinical research on overweight or obese adolescent patients with depression, it was found that aerobic exercise care can effectively improve their negative emotions, processing of things, quality of life, and at the same time improve their physical quality, etc., so as to improve the physical and mental health of the patients. However, there are still some limitations in this study. First, this study selected adolescent depression patients who received consultation in Wuhan Mental Health Center during a specific period of time from June 2019 to June 2022, making the sample correspondingly limited. Second, this study was a retrospective analysis, single-center design study, which could not completely exclude potential confounders and information bias. In future studies, a multi-center research model will be conducted to compensate for the limitations in this study through a more refined study design with a larger sample size. Despite these limitations in this study, this study still provides substantial support for aerobic exercise care for depressed adolescents who are overweight or obese and provides theoretical guidance for clinical practice.

## Conclusion

Aerobic exercise program nursing care for overweight or obese adolescents with depression can effectively improve anxiety and depression, reduce BMI and WHR, and improve social function of patients. On the other hand, such form of management benefits patients with a stable mood, changes their manner of dealing with issues, and improve their quality of life. Therefore, aerobic exercise nursing care has a high nursing care effect on clinical practice and is advocated for popularization and application.

## Availability of Data and Materials

The original contributions presented in the study are included in the article. Further inquiries can be directed to the corresponding author.
